# High-Throughput Interrogation of Ligand Binding Mode Using a Fluorescence-Based Assay**

**DOI:** 10.1002/anie.201202660

**Published:** 2012-06-22

**Authors:** Paweł Śledź, Steffen Lang, Christopher J Stubbs, Chris Abell

**Affiliations:** University Chemical Laboratory, University of CambridgeLensfield Road, Cambridge CB2 1EW (UK) E-mail: ca26@cam.ac.uk

**Keywords:** binding mode, drug discovery, ligand design, protein structures, structure–activity relationships

Structure-based ligand discovery has benefitted from extensive developments in recent years.^1^–^4^ In particular, the application of high-throughput crystallography has vastly accelerated these efforts.^5^ This approach is based on the rapid generation of protein–ligand complexes through the soaking of protein crystals with ligands and subsequent automated X-ray data collection and structure determination. However, suitable soakable crystals are not available for many proteins, especially for those complexes in which protein–protein interactions or structural changes play a role. Consequently, there is a need for more high-throughput and crystallographically independent methods for assessing the binding mode of molecules to select candidates for further analysis.

This has become particularly important to us while developing more potent ligands against the polo-box domain (PBD) of polo-like kinase 1 (Plk1). The PBD domain is responsible for the proper cellular localization of Plk1 through an array of phosphorylation-dependent protein–protein interactions mediated by the phosphopeptide-binding groove.^6^, ^7^ Disrupting these interactions has shown potential as a strategy for anticancer therapy.^8^ The discovery of an auxiliary flexible pocket on the PBD surface, which is involved in binding a peptide derived from polo-box interacting protein 1 (PBIP1), has been recently reported.^9^, ^10^ This flexible hydrophobic pocket recognizes its ligand through hydrophobic interactions of four aromatic residues (Y417, Y421, Y481, and F482) with F71_PBIP1_ and an additional hydrogen bond between the hydroxy group of Y417 and the backbone nitrogen of D72_PBIP1_ (Figure 1 a).^9^

**Figure 1:**
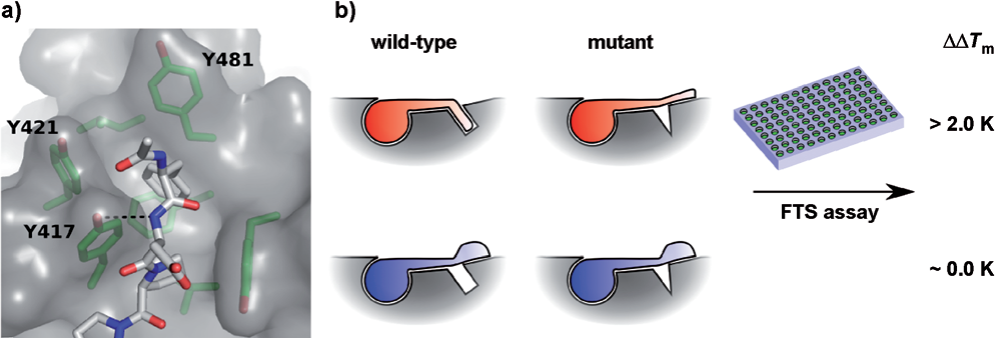
An assay for the hydrophobic pocket binding. a) The hydrophobic pocket on the surface of PBD participates in the binding of the PBIP1 peptide. The hydrogen bond between the phenol of Y417 and D72_PBIP1_ is essential for pocket binding. b) The principle of the use of different protein forms to distinguish the binding modes in a high-throughput fluorescence-based thermal shift (FTS) assay.

Our objective was to prepare more potent ligands of the PBD by improving binding to this auxiliary pocket by optimizing the hydrophobic interactions whilst retaining the key hydrogen bond. To efficiently distinguish specific recognition by the pocket from nonspecific hydrophobic interactions, we adapted a fluorescence-based thermal shift (FTS) assay.^11^ In this assay the ability of a molecule to stabilize the protein during its thermal unfolding is quantified by its thermal shift (Δ*T*_m_): the difference in the protein unfolding temperature in the presence and absence of a ligand. FTS has been successfully applied in fragment and high-throughput screening campaigns;^12^, ^13^ however, it generally provides very limited information on binding and it is not always appropriate for weakly binding ligands.

We designed a procedure through which information on the binding mode can be obtained using FTS. When a modified protein that cannot fully engage a ligand in the selected binding mode is used, ligands adopting such a binding mode are expected to stabilize the mutant protein against thermal unfolding to a lesser extent than the wild-type protein; ligands not engaging in the selected binding mode are expected to bind similarly to both mutant and wild-type protein (Figure 1 b). The difference in thermal shift of a ligand for the wild-type and mutant protein (ΔΔ*T*_m_) is therefore a measure of how beneficial the pocket binding is for a particular ligand. Such an experimental setup eliminates errors arising from the concentration dependence of the assay,^14^ as a single stock solution of the ligand can be used. Also noteworthy is that since in this particular case we were working with elaborated ligands with significant Δ*T*_m_ values, the sensitivity of the assay (Δ*T*_m_>1.0 K) was not a problem and identification of ligands with a significant ΔΔ*T*_m_ value (higher than 2.0 K) was straightforward.

For the proof-of-principle study, we first obtained three different mutants of the PBD to assess their ability to distinguish binding in the hydrophobic pocket (Figure 1 a). The Y417A mutation was expected to prevent the formation of the key hydrogen bond with the ligand. A double mutant with an additional Y421A mutation was also prepared to further reduce the possible scope of hydrophobic interactions.^9^ To assess the specificity of these effects, we prepared a Y481K mutant, which changed the distal end of the pocket, where interaction with the ligand is limited. These mutants were subsequently tested against two PBIP1-derived ligands—FDPPLHSpTA (**1 a**), a peptide that binds in the pocket, and ADPPLHSpTA (**1 b**), which does not. Both the Y417A and Y417A/Y421A mutants exhibited detectable and significant ΔΔ*T*_m_ values for **1 a** (3.7 and 3.2 K, respectively), while ΔΔ*T*_m_ values for **1 b** were within the experimental error (1.2 and 0.1 K). This was further confirmed by the study with the Y481K mutant, which gave negligible ΔΔ*T*_m_ values for **1 a** (−0.8 K) and **1 b** (−1.0 K).

We used the double-mutant Y417A/Y421A to further probe the molecular recognition of the pocket (Table 1). A series of analogues of PBIP1-derived peptide **1 a** with phenylalanine replaced by other acetylated hydrophobic and aromatic amino acids (**2 a**–**f**) were prepared and tested in the FTS binding mode assay. Interestingly, the pocket turned out to be very specific for hydrophobic aromatic residues. Tryptophan-bearing **2 e** exhibited a significant ΔΔ*T*_m_ (3.3 K), indicating hydrophobic pocket binding, in contrast to histidine-bearing **2 f** (ΔΔ*T*_m_=0.5 K). None of tested aliphatic residues (**2 a**–**d**) showed ΔΔ*T*_m_ values higher than 1.0 K.

**Table 1:** The results of the assay for the series of modified peptides (R-DPPLHSpTA-NH_2_). Δ*T*_m_ values are shown for the wild-type (WT) and double-mutant Y417A/Y421A (DM) protein; the difference between them (ΔΔ*T*_m_) and the *K*_D_ value against the wild-type protein is also shown. The color code corresponds to that used in Figure 2 a,b

	R		Δ*T*_m_ [K]		*K*_D_ [nm]
		WT	DM	ΔΔ*T*_m_	
**1 a**	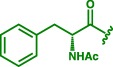	12.9	9.7	3.2	160
**1 b**	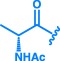	9.8	9.7	0.1	960
**2 a**	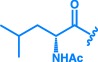	10.3	10.0	0.3	1100
**2 b**	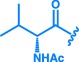	10.9	10.7	0.2	1200
**2 c**	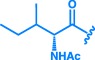	11.5	10.5	1.0	1100
**2 d**	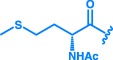	11.0	10.5	0.5	950
**2 e**	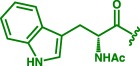	13.6	10.3	3.3	160
**2 f**	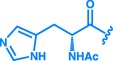	9.3	8.8	0.5	1200
**3 a**	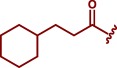	12.5	11.5	1.0	240
**3 b**	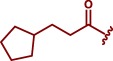	11.6	11.3	0.3	310
**3 c**	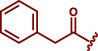	12.2	11.0	1.2	190
**3 d**	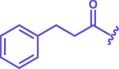	15.4	11.4	4.0	66
**3 e**	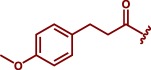	11.6	11.0	0.6	280
**3 f**	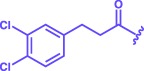	15.8	11.2	4.8	21
**3 g**	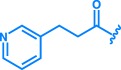	9.9	9.7	0.2	790
**3 h**	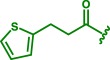	12.7	10.2	2.5	140
**3 i**	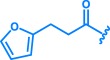	9.9	8.8	1.1	1000
**3 j**	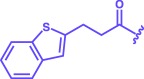	16.0	11.9	4.1	20
**3 k**	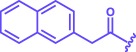	16.0	11.2	4.8	40

To further study the molecular recognition of the pocket, we expanded the series of PBIP1-derived peptides to include non-amino acid N-terminal residues, focusing on, but not limited to, hydrophobic aromatic ring systems (Table 1). As expected, neither of the tested aliphatic ring compounds **3 a**,**b** showed binding in the pocket. Only two out of the four simple phenylalanine analogues **3 c**–**f** gave ΔΔ*T*_m_ values exceeding 2.0 K. Peptide **3 d** with a phenethyl substituent showed slightly better stabilization than the original PBIP1 peptide **1 a**. Two simple analogues of **3 d**, however, showed no indication of hydrophobic pocket binding. Ligand **3 c**, lacking one methylene group, was too short to comfortably insert the phenyl ring to the bottom of the pocket, while the *p*-methoxy substituent in analogue **3 e** appeared to cause a steric clash at the bottom of the pocket. Interestingly, 3,4-dichloro-substituted **3 f** was found to bind well in the hydrophobic pocket. The two *ortho*-chloro substituents in the ring mimic the presence of an additional benzene ring because of their size and hydrophobicity. Three peptides incorporating heterocyclic single-ring systems **3 g**–**j** were also prepared and tested in the assay. Interestingly, the pocket showed significantly higher selectivity for thiophene-derived **3 h** over pyridine-based **3 g** or furan-based **3 j**, even though for **3 h** the observed ΔΔ*T*_m_ value of 2.5 K was not as high as observed for **1 a**. In addition, the two double-ring aromatic systems **3 j**,**k** were tested, both giving high ΔΔ*T*_m_ values indicative of hydrophobic pocket binding. The structures of these compounds suggest that they are able to insert their benzene rings into the pocket, reinforcing the suggestion of its selectivity for aromatic moieties.

Analysis of peptide binding to the protein by isothermal titration calorimetry (ITC; Table 1 and Table S1 in the Supporting Information) revealed a clear correlation between the Δ*T*_m_ for wild-type protein and binding affinity (p*K*_D_) (Figure 2 a). The peptides are clustered into three groups (designated weak, medium, and tight binders), with the initial pocket-binding peptide **1 a** being in the middle group. The correlation between ΔΔ*T*_m_ and *K*_D_ furnishes additional insight into the binding mode (Figure 2 b). Peptides identified in the assay as pocket binders have in general shown higher affinity than those not utilizing the pocket, and all the most potent ligands (**3 d**, **3 f**, **3 j**, and **3 k**, colored purple) also bind to the pocket exhibiting the highest ΔΔ*T*_m_>4.0 K. None of the peptides designated as weak binders (in blue) showed pocket binding. The seven compounds clustered together as medium binders in the center of the plot of p*K*_D_ against Δ*T*_m_ (Figure 2 a) are resolved in the plot of p*K*_D_ against ΔΔ*T*_m_ (Figure 2 b). Peptides **3 a**–**c**, **e** (in red) gain additional binding affinity apparently from pocket-independent hydrophobic interactions, in contrast to similarly potent **1 a**, **2 b**, and **3 h** (in green), shown to be pocket binders. These two different effects responsible for the increased affinity of our ligands can be readily distinguished by ΔΔ*T*_m_ values, allowing for informed binder optimization.

**Figure 2:**
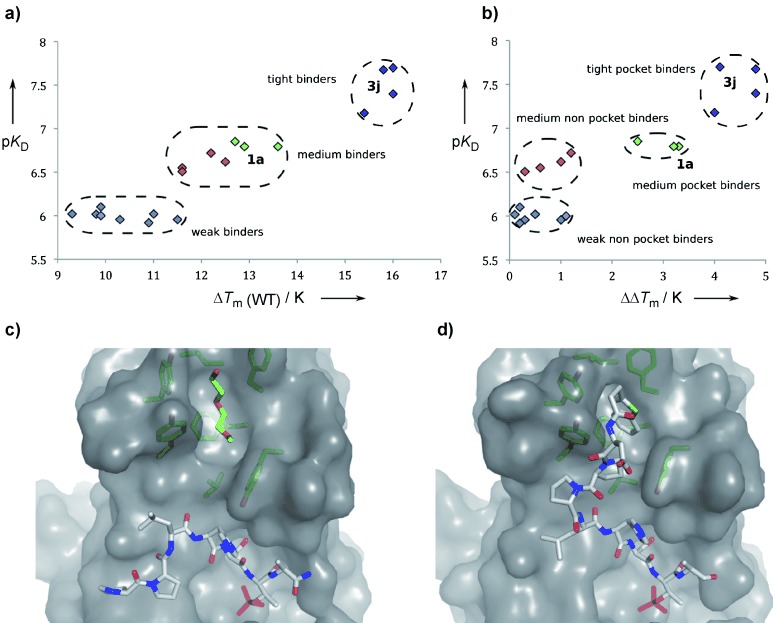
Interpretation and validation of the assay data. a) A plot of p*K*_D_ vs. Δ*T*_m_ shows a correlation between thermal shift and binding affinity; b) a plot of p*K*_D_ vs. ΔΔ*T*_m(WT)_ distinguishes two binding modes leading to increased affinity of modified peptides—pocket-dependent and independent; colors of the data points in (a, b) reflect classification of binders into one of four groups: weak nonpocket (blue), medium nonpocket (red), medium pocket (green), and tight pocket (magenta) and are as in Table 1; c) the structure of **2 a** bound to the protein shows it does not bind to the pocket, which is occupied by PEG300; d) the structure of **3 j** shows that it binds in the pocket, as indicated by the FTS assay.

To confirm the binding modes, we co-crystallized two representative examples with the PBD, the leucine-based peptide **2 a** (ΔΔ*T*_m_=0.3 K) and the most potent ligand **3 j** (ΔΔ*T*_m_=4.1 K). As predicted, peptide **2 a** turned away from the pocket, corroborating the result of the assay (Figure 2 c). In contrast, **3 j** bound in a way similar to **1 a**, retaining the key hydrogen bond and the benzene ring of thiophene was inserted into the pocket, mimicking the phenylalanine of **1 a** (Figure 2 d).

Our FTS-based assay allowed us to rapidly interrogate the binding mode of a series of peptides binding to the PBD of Plk1. The approach is high throughput, as all the FTS experiments described in Table 1 could be performed in duplicate on a single 96-well plate in less than two hours. The results of this assay allowed us to readily observe both the correlation between binding mode and ligand affinity as well as the selectivity of the pocket for particular aromatic moieties. They validate ΔΔ*T*_m_ as a valuable parameter in the structure-based ligand-discovery process, allowing for much deeper insight into structure–activity binding-mode relationships than a purely activity-oriented screen.^15^ As our experimental setup is generalizable, we believe that its adaptation for other targets may be useful, especially for challenging systems like protein interfaces.

## Supplementary material

Detailed facts of importance to specialist readers are published as ”Supporting Information”. Such documents are peer-reviewed, but not copy-edited or typeset. They are made available as submitted by the authors.
