# Supplementary material for: High-Throughput Interrogation of Ligand Binding Mode Using a Fluorescence-Based Assay
Source: Angew Chem Int Ed Engl. 2012 Jun 22;51(31):7680–3. doi: 10.1002/anie.201202660 (PMC3556687; doi:10.1002/anie.201202660)
Supplement: Supplementary file 1 [file anie0051-7680-SD1.pdf]

Angewandte Chemie
Eine Zeitschrift der Gesellschaft Deutscher Chemiker

Supporting Information

© Wiley-VCH 2012

69451 Weinheim, Germany

High-Throughput Interrogation of Ligand Binding Mode Using a Fluorescence-Based Assay**

*Paweł Śledź, Steffen Lang, Christopher J. Stubbs, and Chris Abell**

anie_201202660_sm_miscellaneous_information.pdf

Protein preparation:

The full-length polo-box domain of Plk1 (residues 345-603), its three mutants (Y417A, Y417A/Y421A and Y481K), and the short construct (residues 371-594) were prepared according to previously described procedures (ref. 9 in the main text).

Ligand preparation.

All modified phosphopeptides described in this article were prepared by manual solid phase peptide synthesis (SPPS) using an Fmoc protection group strategy on a NovaBioChem Rink Amide AM resin (100-200 mesh). To increase the throughput of the synthesis and take advantage of ligand similarity first the synthesis of [DPPLHSpTA] fragment of the peptide was performed. Subsequently, the resin was split and the N-terminal residue was added.

Crude peptides were purified by reverse-phase HPLC on a Gilson GX-271 equipped with a Gilson 171 diode array detector using a Polaris C8-A (5 μm; 4.6 x 300 mm (analytical), 21.2 x 300 mm (preparative); Varian, inc.) column at 1 mL/min (analytical) or 21 mL/min (preparative) using a linear gradient of 5 % to 60 % B over 30 min. The solvent system used was A (0.1 % (v/v) TFA in H2O) and B (0.1 % (v/v) TFA in acetonitrile). Peptide identities were confirmed by MALDI-TOF-MS (ABI 4700 Proteomics Analyzer, Applied Biosystems) and amino acid analysis.

FTS – fluorescence-based thermal shift assay.

Thermal shift experiments were performed using a Roche Light Cycler 480 and 96-well plates. The samples were heated in the thermal cycler from 37 to 62 °C with a heating rate of 0.3 °C/min. The thermal unfolding event was observed by adding 2.5x Sypro Orange dye, which was excited at 490 nm and emission was detected at 530 nm. Measurements were performed using a buffer containing 50 mM MES (2-(N-morpholino)ethanesulfonic acid) pH 6.8, 50 mM NaCl and 2 mM EDTA. These conditions were previously optimized for the PBD system (ref. 9 in the main text).

High throughput FTS experiments were conducted in triplicates using a total of 100 μL solution per well, consisting of 90 μL buffered protein solution (1 μM), 10 μL 1 mM peptide solution and 0.05 μL 5000x Sypro Orange solution. Different forms of the protein (wild-type and mutants) were used on a single plate to allow for a direct comparison of observed ΔT_m values and reliable calculation of $\Delta\Delta T_m$ parameter. In such

a setup 15 ligands can be tested in parallel within one-two hours of experimental time if experiments are performed in triplicates (23 if in duplicates and over forty if measurements are taken in singletons).

Isothermal titration calorimetry

ITC experiments were performed using the ITC200 instrument (Microcal Inc. – GE Healthcare) at 25 °C. His_6-PBD345-603 was loaded into the ITC cell at concentration of 30-40 μM. Ligands were dissolved in the same buffer to the concentration of 500 μM. Typically, 30-36 injections of 1.0 μL in volume were done over a period of 30-40 min. Data was fitted to single binding site model using the Origin software package provided by the manufacturer. Thermodynamics parameters for binding of ligands to the WT protein are shown in Supplementary Table 1. Comparison with the binding data for selected ligands to the double mutant (Y417A/Y421A) is shown in Supplementary Table 2.

Crystallization of protein complexes.

PBD-peptide complexes were generated by mixing the protein concentrated to 10-15 mg/mL with the ligand in the molar ratio of 1 : 1.2. These complexes were crystallised in a sitting drop setup by mixing a solution of complex concentrated to 10-15 mg/mL with the well solution in a 1:1 (v/v) ratio. Crystals appeared after 2-7 days. Crystals of PBD-2a complex were grown after 2 days in the solution containing 0.1 M MES pH 6.5 and 30% PEG300 as a precipitant. Crystals of PBD-3j were grown after 7 days in the solution containing 0.1 M sodium acetate, 1.0 M LiCl and 30% PEG 6000 as a precipitant and were of different morphology and significantly lower quality than those of PBD-2a complex. Due to the presence of PEG no further cryoprotection was necessary.

Data collection and structure solution and refinement

Experimental diffraction data were collected at the ID-23-2 beamline of the European Synchrotron Radiation Facility (PBD-2a complex) or in-house using X8 Proteum diffraction system equipped with MICROSTAR rotating anode x-ray generator, Hellios MX optics and Platinum135 CCD detector (PBD-3j complex). Data were processed with XDS[1] or Proteum software package and structures were solved by molecular replacement using Molrep[2] or Phaser[3] from the CCP4 program suite. Models were

manually rebuilt with Coot[4] and structures were refined using Refmac.[5] The summary of data collection and refinement is shown in Supplementary Table 3. Structures were visualized using PyMOL.

References:

[1] W. Kabsch, *Acta Crystallogr. D Biol. Crystallogr.* **2010**, *66*, 125–132.
[2] A. Vagin, A. Teplyakov, *Acta Crystallogr. D Biol. Crystallogr.* **2010**, *66*, 22–25.
[3] A. J. McCoy, R. W. Grosse-Kunstleve, P. D. Adams, M. D. Winn, L. C. Storoni, R. J. Read, *J Appl Crystallogr* **2007**, *40*, 658–674.
[4] P. Emsley, K. Cowtan, *Acta Crystallogr. D Biol. Crystallogr.* **2004**, *60*, 2126–2132.
[5] G. N. Murshudov, A. A. Vagin, E. J. Dodson, *Acta Crystallogr. D Biol. Crystallogr.* **1997**, *53*, 240–255.

Supplementary Table 1. Thermodynamic characterization of ligand binding to PBD by ITC.

R group		N	K_D [nM]	ΔH [kcal/mol]	ΔS [cal/mol·K]
**1a**	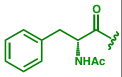	1.00	164 ± 11	-20.2 ± 0.10	-36.6
**1b**	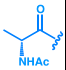	1.01	962 ± 36	-19.7 ± 0.11	-38.4
**2a**	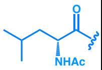	1.04	1099 ± 47	-15.4 ± 0.10	-24.3
**2b**	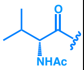	1.00	1212 ± 64	-14.5 ± 0.11	-21.4
**2c**	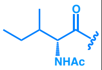	0.96	1078 ± 49	-14.5 ± 0.09	-21.3
**2d**	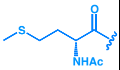	0.98	952 ± 54	-15.8 ± 0.11	-25.5
**2e**	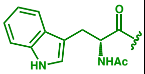	0.99	164 ± 18	-18.6 ± 0.14	-31.2
**2f**	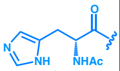	1.01	1172 ± 52	-14.8 ± 0.09	-22.5
**3a**	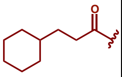	0.99	244 ± 24	-16.8 ± 0.14	-26.1
**3b**	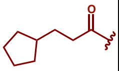	1.02	315 ± 11	-17.1 ± 0.05	-27.7
**3c**	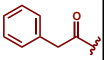	1.03	193 ± 7	-18.5 ± 0.05	-31.1
**3d**	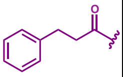	1.05	66 ± 7	-20.3 ± 0.10	-35.2
**3e**	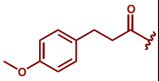	1.04	283 ± 11	-18.5 ± 0.06	-32.0
**3f**	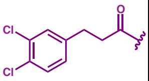	1.00	21 ± 2	-19.8 ± 0.06	-31.3
**3g**	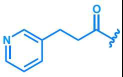	1.03	794 ± 24	-15.5 ± 0.05	-24.1
**3h**	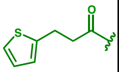	1.00	142 ± 9	-16.5 ± 0.07	-24.1
**3i**	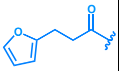	1.01	1034 ± 57	-17.7 ± 0.10	-31.9
**3j**	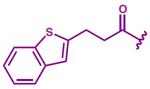	1.01	20 ± 3	-23.3 ± 0.09	-42.8
**3k**	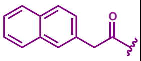	0.99	40 ± 6	-15.4 ± 0.08	-17.9

Supplementary Table 2. Comparison of K_D for wild-type and double-mutant protein binding for selected ligands characterized crystallographically.

R group	K_D [WT] [nM]	K_D [DM] [nM]	K_D [WT]/[DM]
**1a** 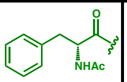	164	720	0.23
**2a** 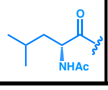	1099	1040	1.06

Supplementary Table 3. Summary of the data collection and refinement data for protein crystals used in the study.

**Ligand:**	**2a**	**3j**
**PDB id**	**4e9c**	**4e9d**
**Data collection**		
X-ray source	ESRF ID 23-2	X8 Proteum
Wavelength (Å)	0.8726	1.5406
Space group	$P2_1$	P1
Cell dimensions		
a, b, c (Å)	35.63 89.78 37.11	35.87 36.30 47.47
α, β, γ (°)	90.0 109.3 90.0	85.8 77.2 68.2
Resolution	44.89 – 1.70	46.29 – 2.75
(high res shell)	(1.80 – 1.70)	(2.85 – 2.75)
Rsym	10.8 (69.3)	14.7 (46.1)
I/σI	11.49 (2.37)	7.41 (2.15)
Completeness (%)	99.0 (96.2)	96.2 (95.8)
Redundancy	3.76 (3.68)	3.42 (3.40)
No. reflections	24 213	5 422
**Refinement**		
Rwork/Rfree	19.2 (23.2)	27.0 (33.0)
PBD molecules in the asymmetric unit	1	1
No. atoms		
Protein*	1884	1833
Ligand/ion	22	6
Water	170	32
RMS deviations		
Bond lengths (Å)	0.007	0.026
Bond angles (°)	1.0	2.8
**Model quality**		
B-factors		
Protein	9.6	13.2
Ligand/ion	29.1	21.1
Water	21.7	3.0
Ramachandran		
Favoured	97.3	95.0
Allowed	2.7	5.0
Outlier	0.0	0.0

* phosphopeptides were treated as protein
